# Fleming’s legacy

**DOI:** 10.1093/jacamr/dlaf167

**Published:** 2025-10-24

**Authors:** R Andrew Seaton

**Affiliations:** Department of Infectious Diseases, Queen Elizabeth University Hospital, NHS Greater Glasgow and Clyde, Glasgow, UK; Scottish Antimicrobial Prescribing Group, Healthcare Improvement Scotland, Glasgow, UK; British Society for Antimicrobial Chemotherapy, Birmingham, UK; School of Medicine, Dentistry and Nursing, University of Glasgow, Glasgow, UK

2028 will mark the centenary of the observation of the inhibitory effects of the mould *Penicillium notatum* (now *chrysogenum*) on the growth of *Staphylococcus aureus* in St Mary’s hospital London by the Ayrshire-born microbiologist Sir Alexander Fleming.^1^ Through work in the Sir William Dunn School of Pathology, University of Oxford by fellow laureates Howard Florey and Ernst Chain, with crucial contributions from Norman Heatley and Edward Abraham, the world’s first antibiotic penicillin was painstakingly extracted, purified and put into clinical use. Soon after, penicillin was transforming the lives of those with serious infections, including World War II casualties with infected wounds. This great discovery, the impetus for so many other antibiotic discoveries, heralded the golden era of modern medicine. Penicillin and future antibiotics were not only curing a wide range of bacterial infections but creating a scaffold to support so many medical innovations. Routine and emergency surgeries, including caesarean section and joint replacement surgery, were made safe and cancer therapy and transplant medicine became possible. Without penicillin and the subsequent antibiotic discoveries, much of modern healthcare as we know it would not be possible.

In his Nobel Lecture in December 1945, Fleming warned ‘The time may come when penicillin can be bought by anyone in the shops. Then there is the danger that the ignorant man may easily underdose himself and by exposing his microbes to non-lethal quantities of the drug make them resistant’.^2^ Fast forward 60 years, and unregulated availability and indiscriminate antibiotic use had become an everyday reality and antimicrobial resistance (AMR) well established although often poorly appreciated by clinicians and the public. The WHO Global Action Plan on AMR in 2015 called for urgent action including funding to improve surveillance, public awareness and responsible antibiotic use. At the same time the UK AMR Review, commissioned by the UK Government in 2014 and Chaired by Lord Jim O’Neill^3^ estimated that by 2050, up to 90% of all deaths related to AMR will come from those countries least resourced in Africa and Asia. Recognizing that the burden of AMR weighs heaviest in low- and middle-income countries, the UK Government developed the eponymous ‘Fleming Fund’ underlining the UK’s pivotal role in antibiotic discovery, advocacy and commitment to supporting those countries in greatest need. The Fleming Fund has been a relatively small but crucial component of the UK’s overseas aid programme, strengthening AMR surveillance and multi-professional training through a portfolio of grants, global projects and fellowship schemes across 25 countries in Africa and Asia.^4^

The UK Government’s recent announcement to retire the Fleming Fund has come at a critical juncture with estimates suggesting if AMR is left unchecked, there will be up to 10 million AMR associated deaths per year with an estimated US $1.7 trillion loss of global Gross Domestic Product and an additional US $175 billion required for healthcare spending by 2050.^5^ Ending this support is also at odds with the UK’s 5 year National Action Plan to combat AMR where there is a commitment to international AMR diplomacy and support for those in resource poor settings.^6^

Alexander Fleming could not have foreseen the transformational impact of his 1928 discovery on the landscape of modern medicine, nor could he have envisaged the potential unravelling of these advances by the uncontrolled growth in AMR. Although he certainly would have recognized the challenge of grossly infected war wounds reliant on extensive surgical debridement as now witnessed in Eastern Europe^7^ and the Middle East,^8^ it is unlikely he could have anticipated the extent to which these infections have been rendered otherwise untreatable due to multidrug-resistant pathogens. Fleming would have appreciated the false economy of disinvesting in initiatives to embed better practice, knowledge and understanding of AMR in those countries where the need is most acute.

The centenary of the discovery of penicillin should be a cause for celebration and acknowledgement of the pivotal role this discovery has had on modern medicine worldwide. Strong commitment and actions to safeguard antibiotics, through global initiatives supporting education, surveillance and research would be a fitting legacy for Alexander Fleming.
